# Supplementary material for: Percolation transition prescribes protein size-specific barrier to passive transport through the nuclear pore complex
Source: Nat Commun. 2022 Sep 1;13:5138. doi: 10.1038/s41467-022-32857-1 (PMC9437005; doi:10.1038/s41467-022-32857-1)
Supplement: Supplementary file 1 — Supplementary Information [file 41467_2022_32857_MOESM1_ESM.pdf]

Supplementary Information: Percolation transition prescribes protein size-specific barrier to passive transport through the nuclear pore complex.

David Winogradoff[1,2], Han-Yi Chou[1], Christopher Maffeo[1,3] & Aleksei Aksimentiev[1,2,3,*]

[1]*Department of Physics, University of Illinois at Urbana-Champaign, Urbana, IL 61801, USA.*

[2]*Center for the Physics of Living Cells, University of Illinois at Urbana-Champaign, Urbana, IL 61801, USA.*

[3]*Beckman Institute for Advanced Science and Technology, University of Illinois at Urbana-Champaign, Urbana, IL 61801, USA.*

These authors contributed equally: David Winogradoff and Han-Yi Chou.

[*] *email: aksiment@illinois.edu*

This PDF contains:

Suppl. Fig. 1	Density and stoichiometry of FG-nups across NPC models	S3
Suppl. Fig. 2	Properties of the FG-nup mesh	S4
Suppl. Fig. 3	Passive diffusion across the NPC	S5
Suppl. Fig. 4	Protein localization in CG simulations of passive diffusion	S6
Suppl. Fig. 5	Potentials of mean force extracted from CG simulations	S7
Suppl. Fig. 6	Protein localization in CG simulations of passive diffusion	S8

Suppl. Fig. 7 Potentials of mean force extracted from CG simulations of passive diffusion (25 nm) S9
Suppl. Fig. 8 Passive diffusion across the Lin2016 model of the NPC . . . S10
Suppl. Fig. 9 Molecular mass versus geometric radius S11
Suppl. Fig. 10 PMF barrier from void analysis of the FG-nup mesh S12
Suppl. Fig. 11 Void model of the translocation barrier S13
Suppl. Fig. 12 Mean first-passage time from the 1D Fokker-Planck model . S14
Suppl. Fig. 13 Fokker-Planck model of passive diffusion through the NPC S15
Suppl. Fig. 14 Comparison to results from previous studies of passive diffusion S16
Suppl. Fig. 15 Comparison of FG-nup densities of two yeast NPC models. S17
Suppl. Fig. 16 Validation of the coarse-grained model implementation . . S18
Suppl. Fig. 17 Scaling behavior of the FG-nups radius of gyration S19
Suppl. Fig. 18 Structural fluctuations of globular proteins in all-atom MD simulations S20
Suppl. Fig. 19 Effect of local diffusion model on Fokker-Planck MFPT . . S21
Suppl. Table 1 Rigid-body approximation of protein size and diffusion . . . S22
Suppl. Table 2 The radius and the diffusion constants of each spherical probe used for void analysis and Fokker-Planck calculations S23
Suppl. Table 3 FG-nup domains of the Kim2018, Kim2018+ and Huang2020 NPC models. S24
Supplementary Methods S25
Supplementary References S27

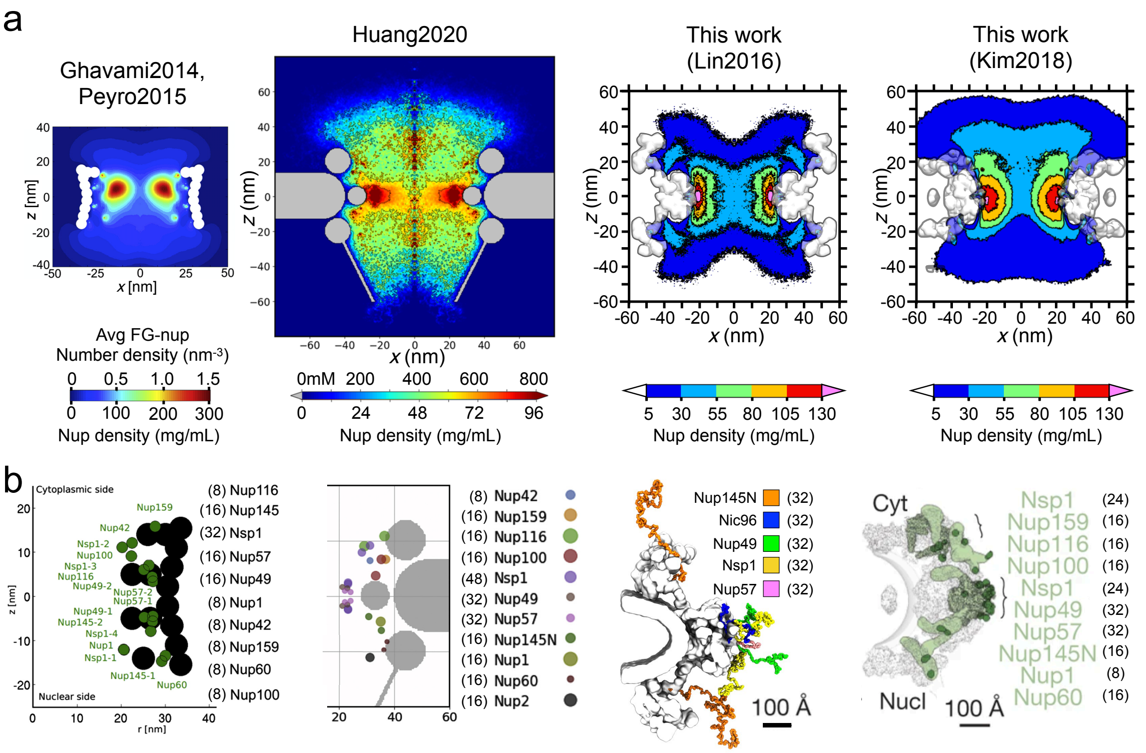

Supplementary Fig. 1: Density and stoichiometry of FG-nups across recent computational models of NPC. a To-scale comparison of the average FG-nup densities from the previous computational studies of Ghavami et al. [1] (the same model is also used in Peyro et al. [2]) and of Huang et al. [3], to the densities resulting from our CG simulations of the Lin2016 and Kim2018 models. To enable direct comparison of the density maps, we converted the FG-nup number density scale (in nm^{-3}) used by Ghavami et al. and the amino-acid concentration scale (in mM) used by Huang et al. to mg/mL assuming that each amino-acid residue has a molecular weight of 120 Da. The mg/mL scale is listed below each original color bar. **b** Stoichiometric information for each NPC model, including scaffold shape, anchor position and copy-number of each FG-nup species. Left-most panels adapted with permission [1]; Copyright 2014, Elsevier. Second from the left panels adapted with permission [3]; Copyright 2020, Elsevier. Bottom right panel adapted with permission [4]; Copyright 2018, Springer Nature.

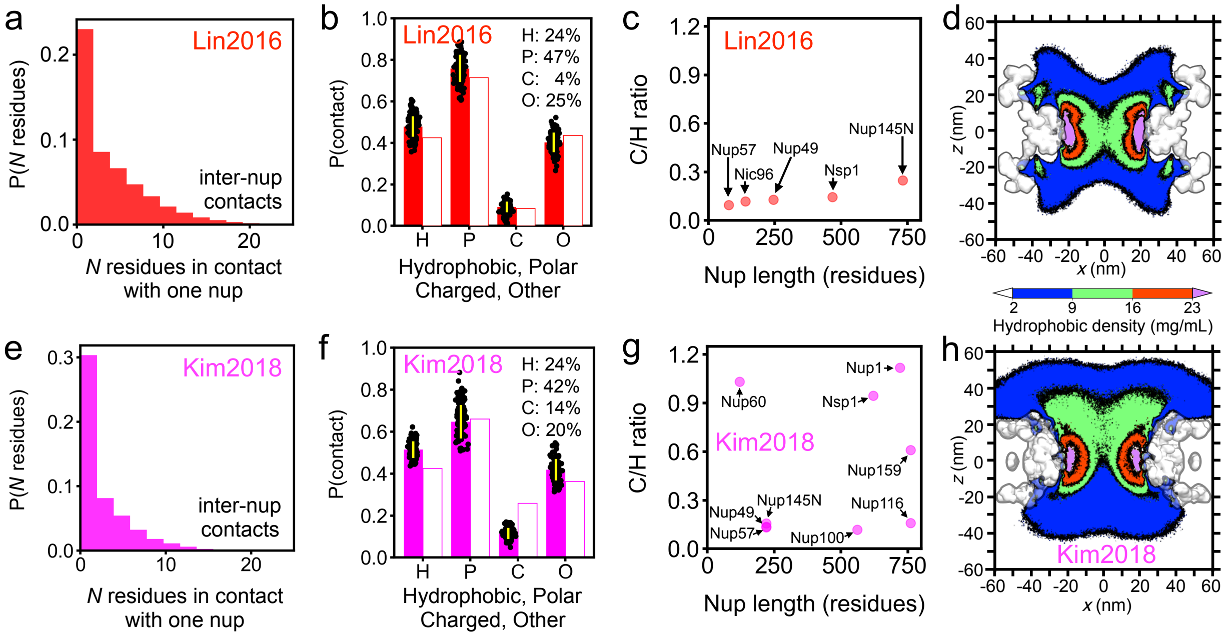

Supplementary Fig. 2: Properties of the FG-nup mesh. **a** The probability of an FG-nup forming contacts with N residues of any other FG-nup as seen in our CG simulations of the Lin2016 NPC model. Data based on N = 13,000 frames. **b** The probability of a residue type being involved in an inter-chain contact (filled bars). For reference, we plot the fraction of contacts expected to include a specific residue type for a random mixture of residues of the same composition as the FG-nup mesh, i.e., $F_\mathrm{X} = P_{\text{all including X}}/(P_\mathrm{H}P_\mathrm{H} + P_\mathrm{H}P_\mathrm{P} + P_\mathrm{P}P_\mathrm{H} + P_\mathrm{H}P_\mathrm{C} + P_\mathrm{C}P_\mathrm{H} + P_\mathrm{H}P_\mathrm{O} + P_\mathrm{O}P_\mathrm{H} + P_\mathrm{P}P_\mathrm{P} + P_\mathrm{P}P_\mathrm{C} + P_\mathrm{C}P_\mathrm{P} + P_\mathrm{P}P_\mathrm{O} + P_\mathrm{O}P_\mathrm{P} + P_\mathrm{C}P_\mathrm{C} + P_\mathrm{C}P_\mathrm{O} + P_\mathrm{O}P_\mathrm{C} + P_\mathrm{O}P_\mathrm{O})$, where the fraction of residues of type X in the FG-nup mesh $P_\mathrm{X} = N_\mathrm{X}/(\Sigma_{\mathrm{H,P,C,O}} N_i)$, N_X is the number of residues of type X in the mesh; X and Y denote either H, P, C or O. The relative abundance (percentage) of residues of each type is specified in the graph. Data presented as mean values ± SD, based on frames binned into N = 240 groups. **c** The charge-to-hydrophobicity ratio of each FG-nup segment in our Lin2016 model as a function of the nup fragment length. Points annotated by nup species name. **d** Average density of the hydrophobic residues within the FG-nup mesh of our Lin2016 model. The density was averaged over the respective CG trajectories and along the y coordinate within the $[-7.5, +7.5]$ nm range. **e–h** Same as in panels a–d but for the Kim2018 model. The residues were categorized as hydrophobic, H (Ala, Val, Ile, Leu, Phe, Trp), polar, P (Ser, Thr, Asn, Gln, His), charged, C (Arg, Lys, Asp, Glu) or other, O (Met, Tyr, Gly, Pro, Cys). Data in panel e based on N = 6,500 frames. Data in panel f presented as mean values ± SD, based on frames binned into N = 240 groups. The contact analysis of the Lin2016 model was performed on the final 6,500 μs fragments of the two CG equilibration trajectories, sampled every 0.1 μs. The contact analysis of the Kim2018 model was performed on the final 6,500 μs fragment of the CG equilibration trajectory, sampled every 0.1 μs. A contact was determined to exist when the distance between two amino acid beads from different nups was less than 0.8 nm.

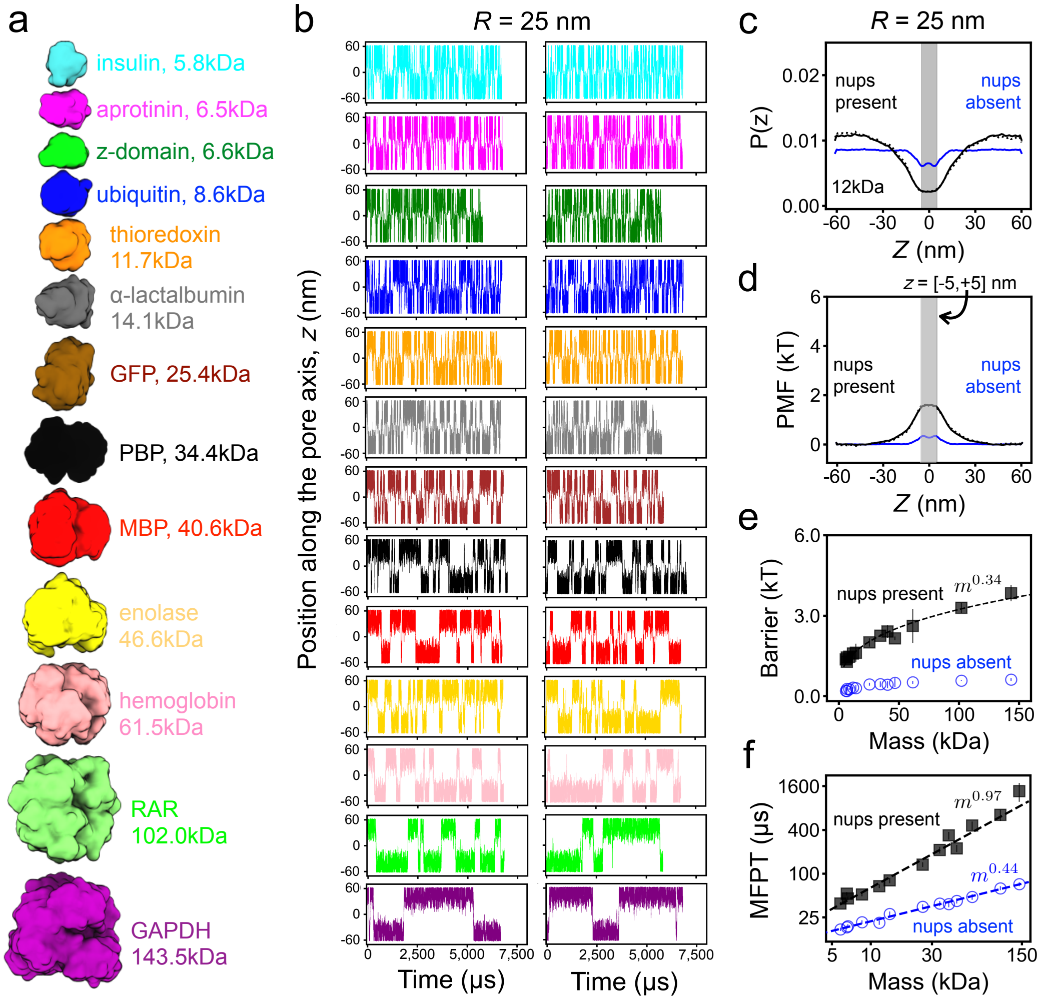

Supplementary Fig. 3: Passive diffusion across the NPC. **a** To-scale structural representation of all proteins used for CG simulations of passive diffusion. **b** Center-of-mass z coordinate of each protein (colors defined in panel a) versus simulation time. The simulations traces in the two columns differ by the initial placement of the protein. The simulations were performed in the presence of a 25 nm-radius confinement potential. **c** Normalized distribution of the CoM z coordinate of thioredoxin. The black dotted and solid lines show the distribution extracted directly from the simulations and the symmetrized distribution, respectively. The blue line shows a symmetrized distribution for the simulation carried out in the absence of the FG-nup mesh. **d** Potential of mean force (PMF) for thioredoxin transport across the NPC. A PMF barrier is defined as the average value within $|z| < 5$ nm. **e** PMF barrier versus protein molecular mass determined from CG simulations of protein diffusion through our complete NPC model (black squares) and the model devoid of all FG-nups (blue circles). Data presented as mean values ± the average point-by-point difference of the unsymmetrized PMF values from $-50 < z < 0$ nm and $0 < z < 50$ nm intervals. PMF curves derived from $N = 140{,}000$ frames. $N = 10$ points along those curves defined the mean barrier, and $N = 100$ points defined the average point-by-point difference. Line shows a power law fit to the data. **f** Mean first-passage time (MFPT) versus protein molecular mass. Note the logarithmic scale of both axes. Power-law fits are shown as dashed lines in panels e and f. In panel f only, the power-law fit to the "nups present" data included proteins up to 62 kDa (hemoglobin). Compared to the 50 nm-radius confinement data, (Fig. 2h), the MFPT values are a factor 3 higher because of the higher effective concentration of the protein.

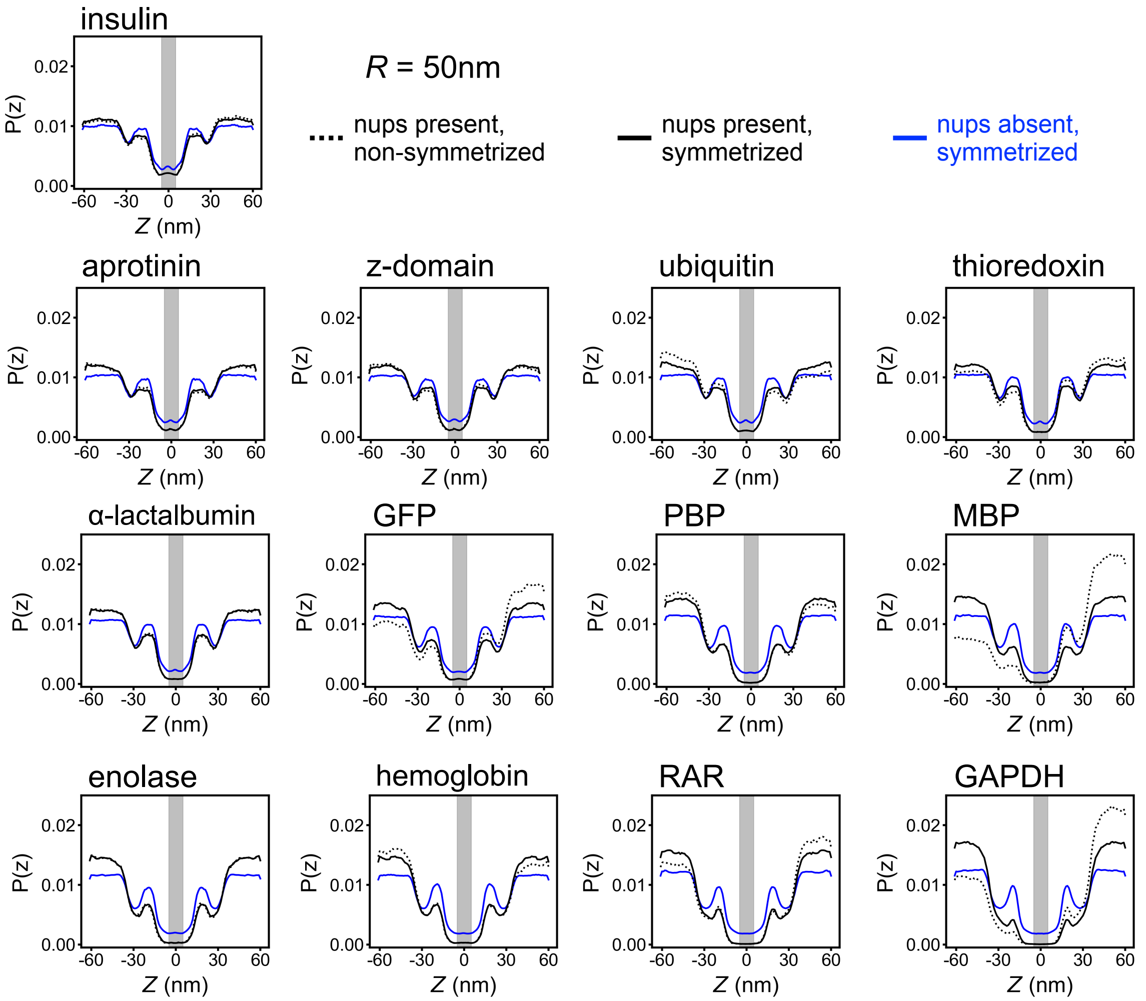

Supplementary Fig. 4: Protein localization in CG simulations of passive diffusion. Each panel shows a normalized distribution of the protein's CoM z coordinate. The black dotted lines show the distributions extracted directly from the CG simulations whereas the black solid lines show the same distributions symmetrized with respect to z=0. The blue lines show symmetrized distributions for the simulation carried out in the absence of the FG-nup mesh. All data derive from the simulations carried out under a 50 nm-radius confinement potential.

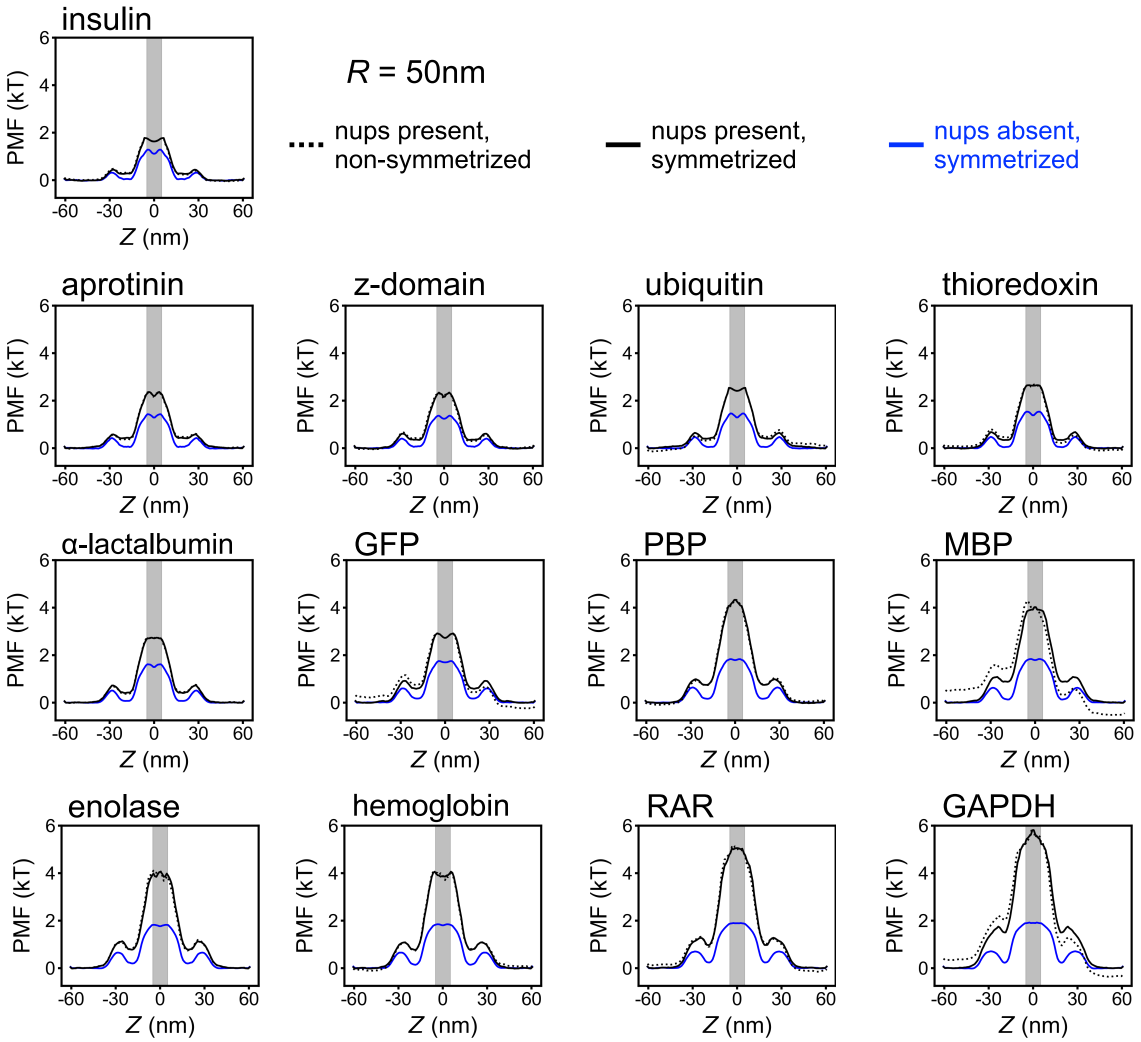

Supplementary Fig. 5: Potentials of mean force for thirteen protein species extracted from CG simulations of passive diffusion. The black dotted lines show the PMFs obtained through Boltzmann inversion of the non-symmetrized CoM distributions, (Supplementary Fig. 4). The solid black lines show the same PMFs symmetrized with respect to $z = 0$. The blue lines show symmetrized PMFs obtained from the simulations carried out in the absence of the FG-nup mesh. The grey rectangles illustrate our definition of a PMF barrier. All data derive from the simulations carried out under a 50 nm-radius confinement potential.

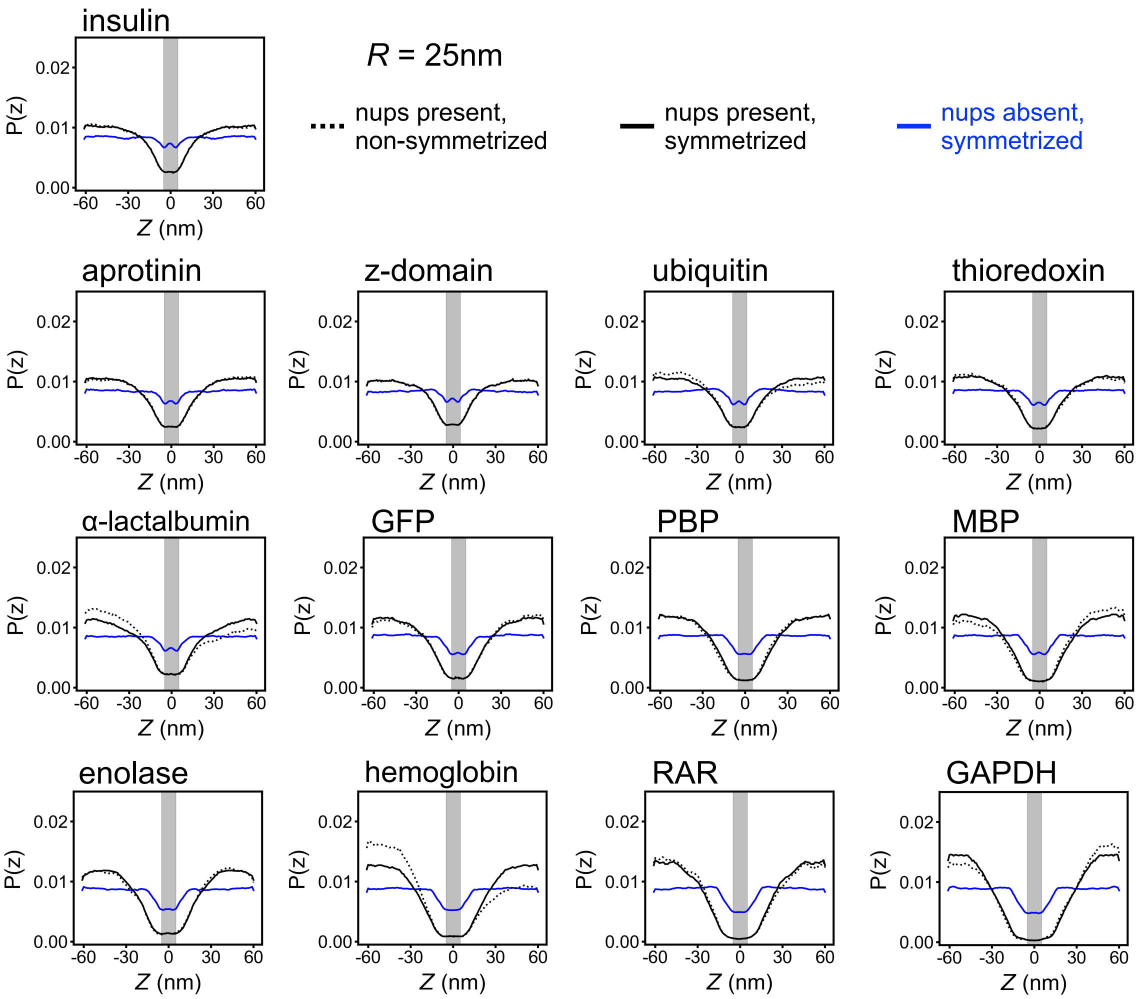

Supplementary Fig. 6: Protein localization in CG simulations of passive diffusion. Each panel shows a normalized distribution of the protein's CoM z coordinate. The black dotted lines show the distributions extracted directly from the CG simulations whereas the black solid lines show the same distributions symmetrized with respect to z=0. The blue lines show symmetrized distributions for the simulation carried out in the absence of the FG-nup mesh. All data derive from the simulations carried out under a 25 nm-radius confinement potential.

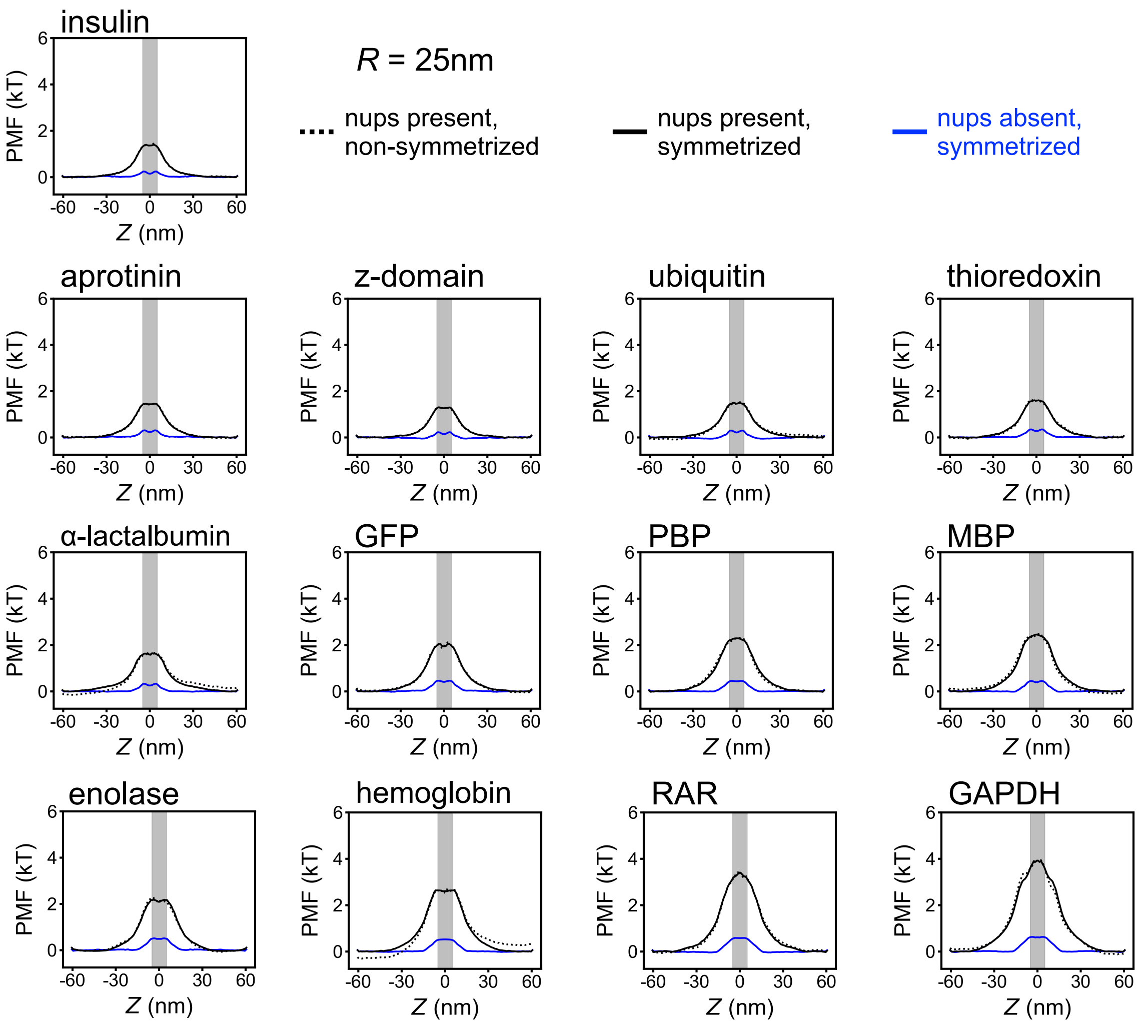

Supplementary Fig. 7: Potentials of mean force for thirteen protein species extracted from CG simulations of passive diffusion. The black dotted lines show the PMFs obtained through Boltzmann inversion of the non-symmetrized CoM distributions, (Supplementary Fig. 6). The solid black lines show the same PMFs symmetrized with respect to $z = 0$. The blue lines show symmetrized PMFs obtained from the simulation carried out in the absence of the FG-nup mesh. The grey rectangles illustrate our definition of a PMF barrier. All data derive from the simulations carried out under a 25 nm-radius confinement potential.

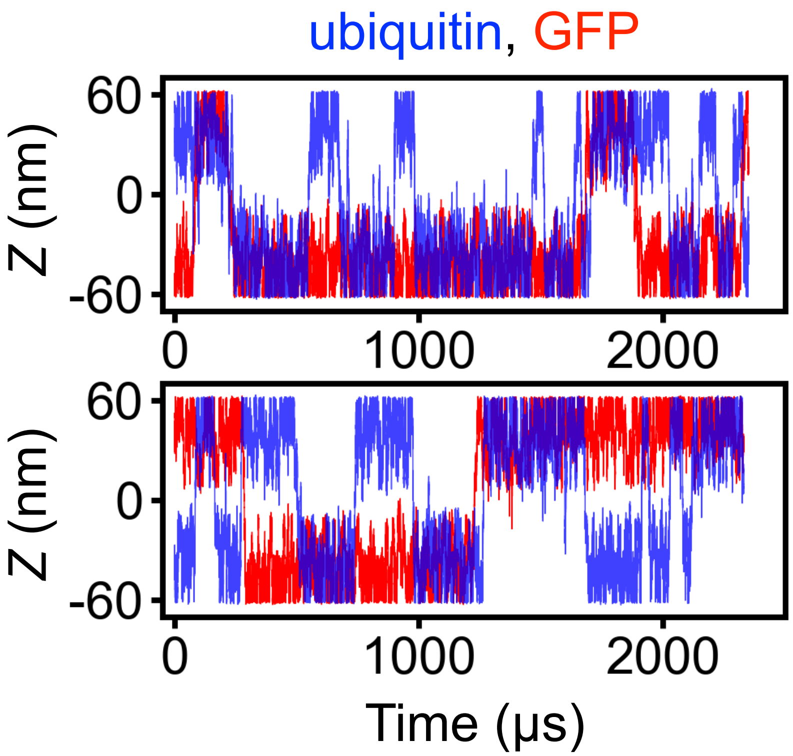

Supplementary Fig. 8: Simultaneous passive diffusion of ubiquitin and GFP across the Lin2016 model of the NPC. Center-of-mass z coordinate of each protein is plotted versus simulation time. The simulations traces in the two rows differ by the initial placement of the two proteins. The simulations were performed in the presence of a 50 nm-radius confinement potential.

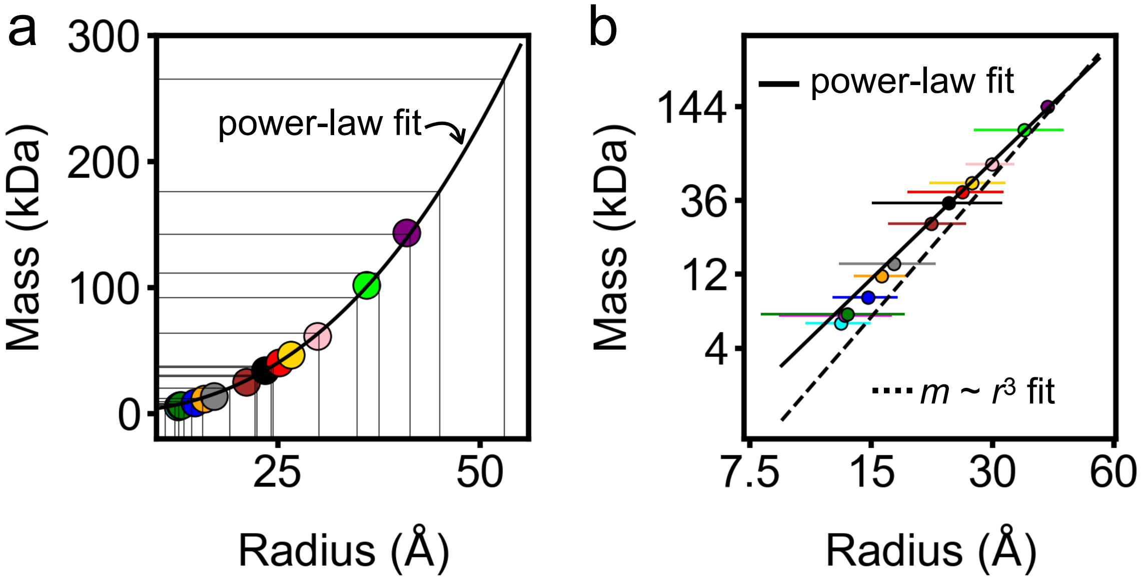

Supplementary Fig. 9: Molecular mass versus geometric radius of the proteins considered in this work. Each colored circle represents one protein species simulated using our CG approach. Panels a and b show the same data using linear (panel a) and logarithmic (panel b) scale for the axes. The radius of a protein was computed by matching the protein's moments of inertia with those of an ellipsoid and then approximating the ellipsoid as a sphere of the same volume. The thick solid line shows the best power-law fit to the data, $\sim r^{2.51}$. The dashed line in panel b also shows an $\sim r^3$ fit for comparison. While one would intuitively expect the protein mass to scale as a cube of the protein radius, we empirically found a slower-growing dependence, which we associate with the presence of voids within the proteins. We used this empirical dependence to associate the radius of a spherical probe with a protein mass. Thus, the thin vertical lines represent the radii of the spherical probes used for the void analysis. The thin horizontal lines indicate the equivalent molecular mass for each spherical probe estimated using the best power-law fit. The raw data along with a mathematical formulation of the geometric radius are provided in (Supplementary Table 1).

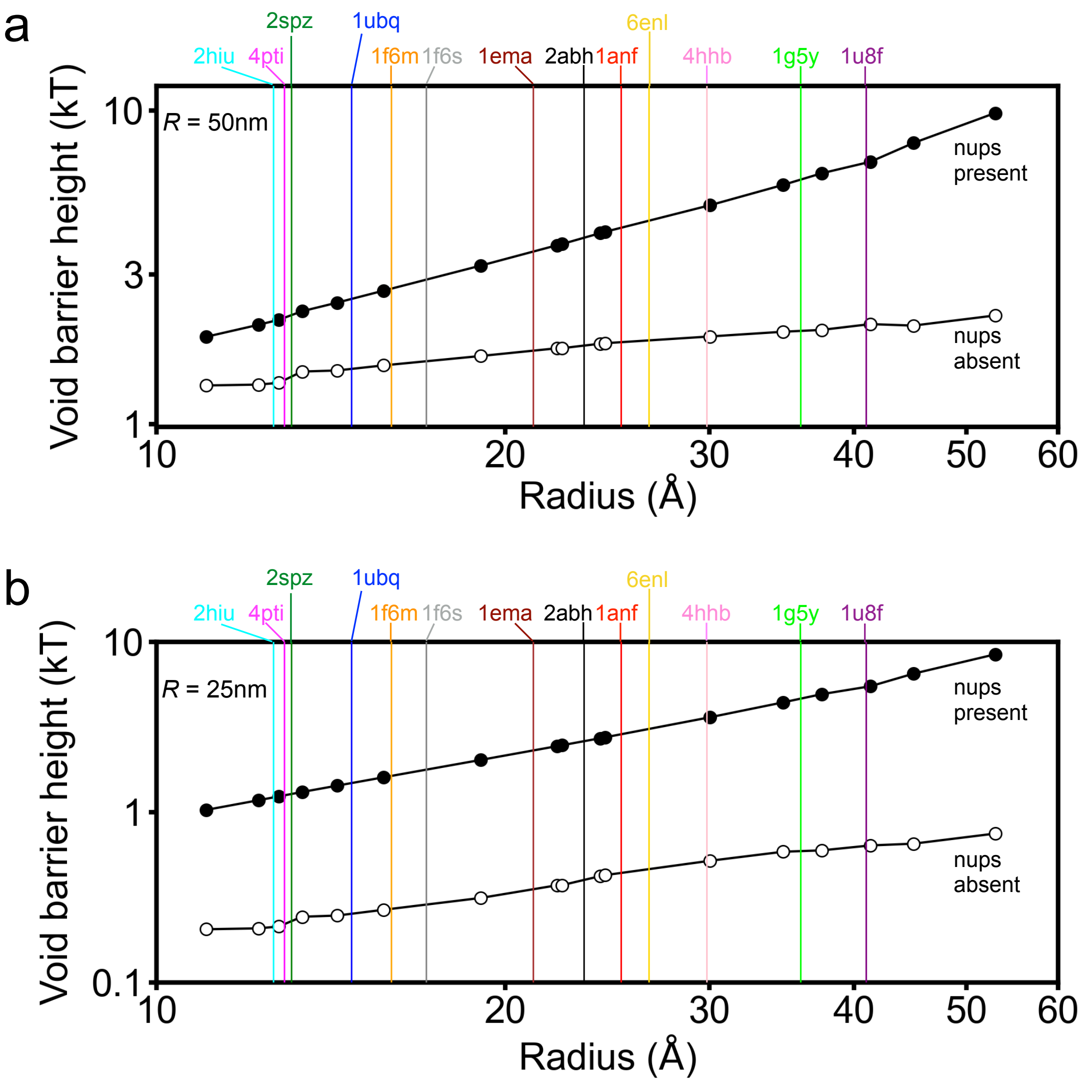

Supplementary Fig. 10: PMF barrier from void analysis of the FG-nup mesh. **a** Height of the PMF barrier versus the radius of the spherical probe used for the void analysis calculations of the translocation PMF. Filled and open circles indicate data obtained for a complete NPC model and a model devoid of the FG-nup mesh, respectively. Vertical lines indicate geometric radii of the proteins used in our CG simulations; each line is annotated with a corresponding PDB ID. The void analysis value of the PMF barrier for each of the thirteen proteins was determined by interpolation of the void analysis data. Note the logarithmic scale of both axes. These data were obtained assuming a 50 nm-radius confinement potential. **b** Same as in panel a, but for a 25 nm-radius confinement potential.

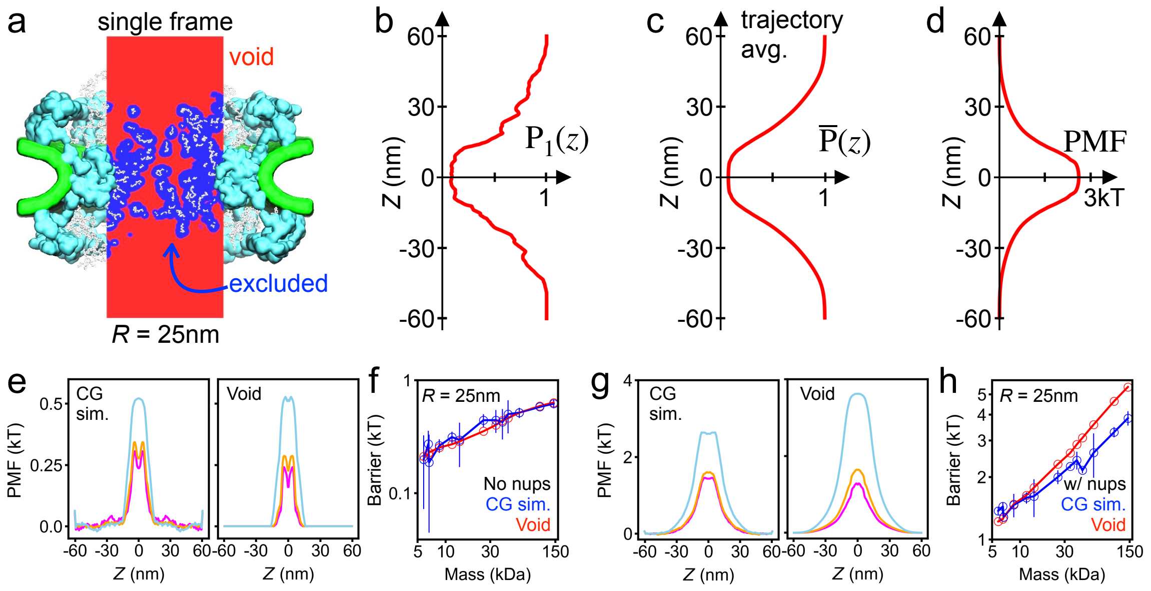

Supplementary Fig. 11: Void model of the translocation barrier. a Void analysis map of an instantaneous NPC configuration computed using a spherical probe of 22.4 Å radius. The volume available to accommodate the probe (void) is shown in red, the volume excluded in blue, FG-nups in white, the scaffold in cyan and the lipid bilayer in green. The image shows a 2D section of a 3D map. **b** The fraction of the NPC volume that can accommodate the probe without clashes as a function of the pore axis coordinate. The fraction was computed by splitting the void analysis map into cylindrical segments of 25 nm radius and 0.6 nm height, coaxial with the pore. The data shown were computed for the instantaneous NPC configuration displayed in panel a. **c** Trajectory-averaged probability of accommodating the probe as a function of the pore axis coordinate, $\overline{P}(z)$, computed by averaging instantaneous void analysis maps over the last 6 ms of the NPC equilibration trajectory, sampled every 1.0 μs. **d** PMF of the spherical probe derived by void analysis. **e** Symmetrized PMF of three protein species (aprotinin, magenta; thioredoxin, orange; and hemoglobin, light blue) derived from brute force CG simulations (left) and of the three spherical probes of approximately the same radius (R_p = 12.75, 15.71 and 30.04 Å) derived from void analysis (right). **f** PMF barrier versus protein mass. Interpolation was used to find void analysis PMF barriers for the proteins simulated using the CG method, (Supplementary Fig. 10). Lines are guides to the eye. Both axes use logarithmic scale. **g,h** Same as in e,f but for the complete NPC model (including nups).

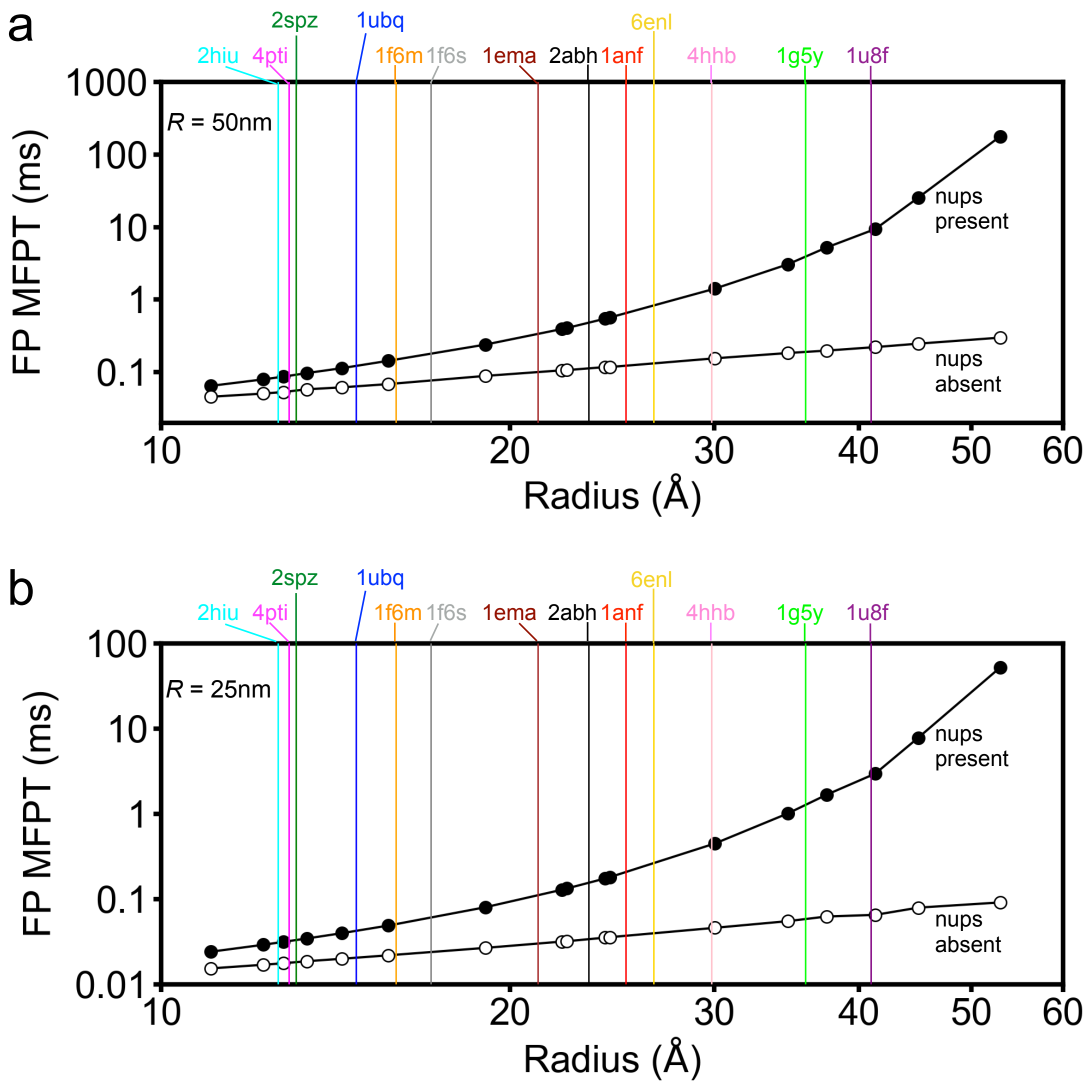

Supplementary Fig. 12: Mean first-passage time from the 1D Fokker-Planck model. a Mean first-passage time versus the radius of the spherical probe used for the void analysis/Fokker-Planck calculations. Filled and open circles indicate data obtained for a complete NPC model and a model devoid of the FG-nup mesh, respectively. Vertical lines indicate geometric radii of the proteins used in our CG simulations; each line is annotated with a corresponding PDB ID. The Fokker-Planck value of the mean first-passage time for each of the thirteen proteins was determined by interpolation of the Fokker-Planck data. Note the logarithmic scale of both axes. These data were obtained assuming a 50 nm-radius confinement potential. **b** Same as in panel a, but for a 25 nm-radius confinement potential.

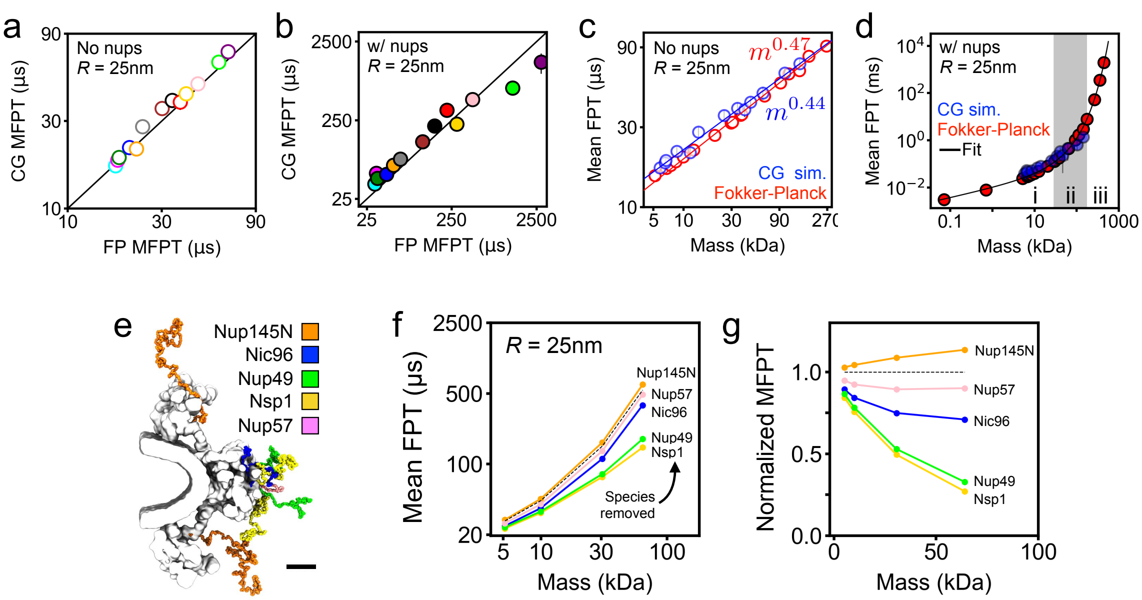

Supplementary Fig. 13: Fokker-Planck model of passive diffusion through the NPC. a,b Comparison of the mean first-passage times (MFPT) calculated from CG simulations to those from our Fokker-Planck void analysis model for nup-less (panel a) and complete (panel b) NPC models. The black line indicates perfect agreement. Data in panels a, b presented as mean values ± SEM, based on traces involving N = 140,000 frames. **c** MFPT from CG simulations (blue) and using our Fokker-Planck approach (red) as a function of protein molecular mass. Note the logarithmic scale of the axes. Power law fits, and their slopes, are specified in the figure. **d** Same as in panel c but for a complete NPC model, with all FG-nups present. The black line is a single fit to all of the Fokker-Planck data, functional form provided in the main text. The three regions (i, ii, iii) correspond to power-law, transition and exponential scaling behavior. **e** Location of each FG-nup species in one sixteenth of the CG model. Black scale bar, 10 nm. **f** MFPT versus molecular mass for an NPC model devoid of one FG-nup species (colors) and with all FG-nups present (dashed black line). **g** MFPT for the deletion mutants normalized by the all species present MFPT. All data in this figure were obtained under a 25 nm radius confinement potential. Interpolation was used to express the results of the Fokker-Planck void analysis model in terms of molecular mass, (Supplementary Fig. 12).

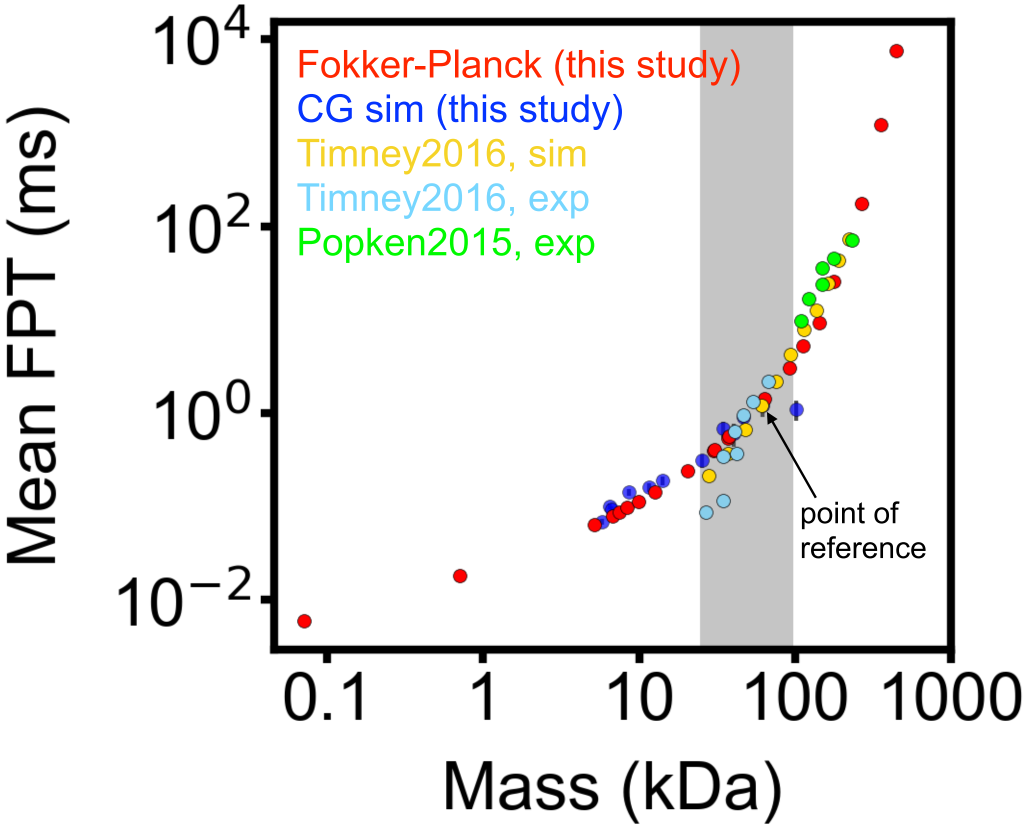

Supplementary Fig. 14: Comparison to results from previous studies of passive diffusion. The mean first-passage time (MFPT) data obtained using our Fokker-Planck approach (red) and our coarse-grained simulations (blue) are plotted as a function of molecular mass along with the simulation data reported by Timney et al. [5] (gold), experimental values from Timney et al. (light blue) and experimental values from Popken et al. [6] (green). To enable quantitative comparison, the MFPT values from Timney et al. [5] and Popken et al. [6] were multiplied by a single constant value: the ratio of the hemoglobin's (61.5 kDa) MFPT obtained from our CG simulations and the MFPT reported by Timney et al. for a sphere of a 61 kDa mass. Note the logarithmic scale of both axes.

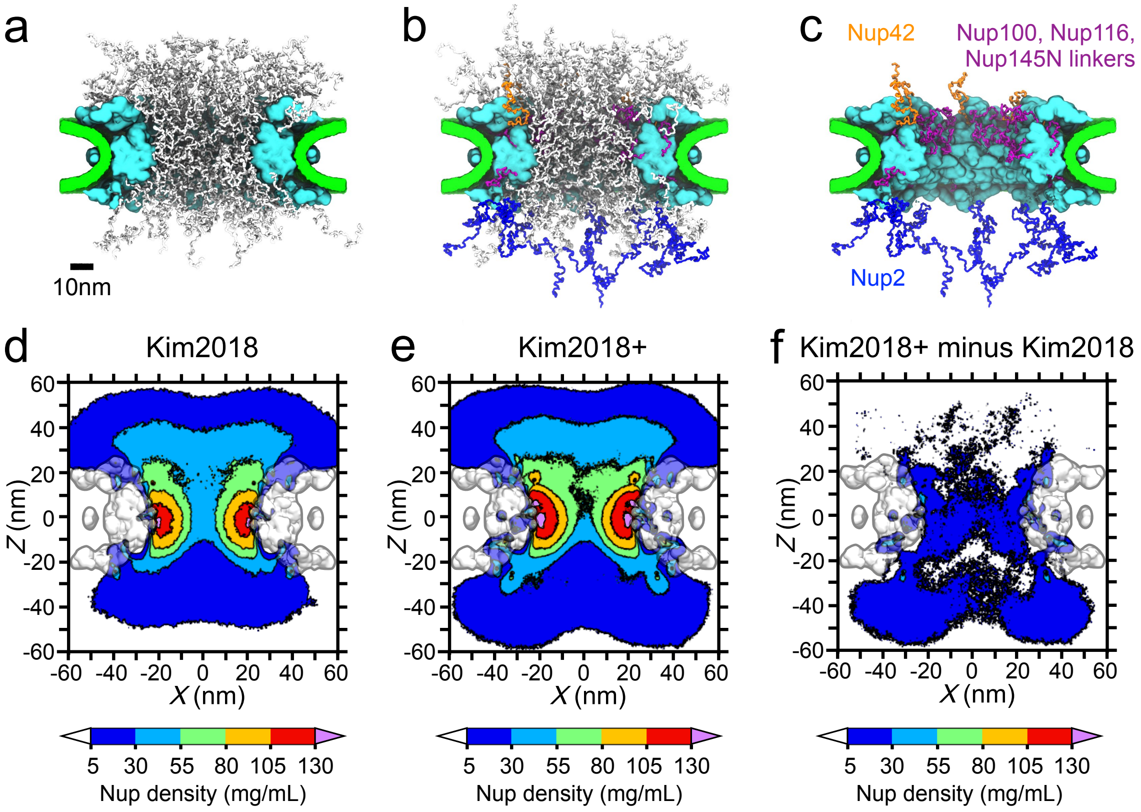

Supplementary Fig. 15: Comparison of FG-nup densities of two yeast NPC models. **a,b** Representative configurations of two separate coarse-grained models of a yeast NPC based on the integrative structure by Kim et al. [4], which we refer to as Kim2018 and Kim2018+. **c** FG-nup domains present in our Kim2018+ model, but absent from the Kim2018 model. A detailed accounting of all FG-nup domains present in these two models is provided in Supplementary Table S3. **d** Cross section of the FG-nup amino acid density of the Kim2018 model averaged over the respective simulation trajectory and along the y coordinate from (-7.5, +7.5) nm. **e** Same as in panel d but for the Kim2018+ model. **f** The difference between Kim2018+ and Kim2018 density maps.

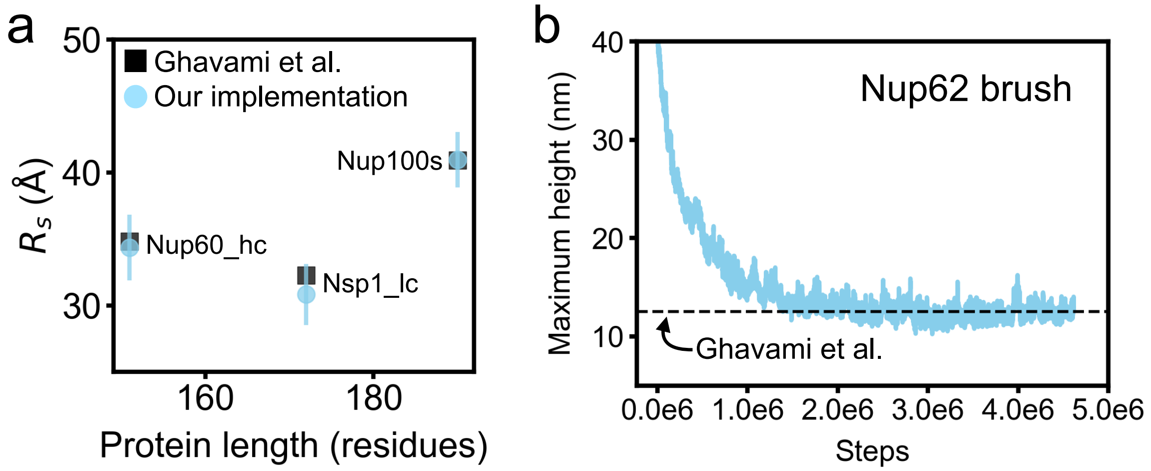

Supplementary Fig. 16: Validation of the coarse-grained model implementation. a Average Stokes radius, R_s, of isolated FG-nups as a function of the number of amino acid residues comprising the FG-nups. Black squares indicate results of Ghavami et al. [1]. Blue dots are the average values from our simulations performed using our implementation of the same coarse-grained model; error bars show one standard deviation. Following the nomenclature used in Ref. 1, "hc" stands for high charge, "lc" for low charge and "s" for stalk. Data presented as mean values $\pm$ SD, based on trajectories involving $N = 1{,}001$ frames each. **b** Maximum height of an FG-nup brush calculated from a test simulation performed using our implementation of the CG model. Dashed line (13 nm) indicates the convergent value from Ref. 1. To create the brush, Nup62 fragments (residues 1-240) were grafted onto a flat surface in a 10×10 array, with the anchor points being 2.4 nm apart. For each simulation frame, the residue of the entire brush located farthest from the flat surface was used to compute the "maximum height" of the brush.

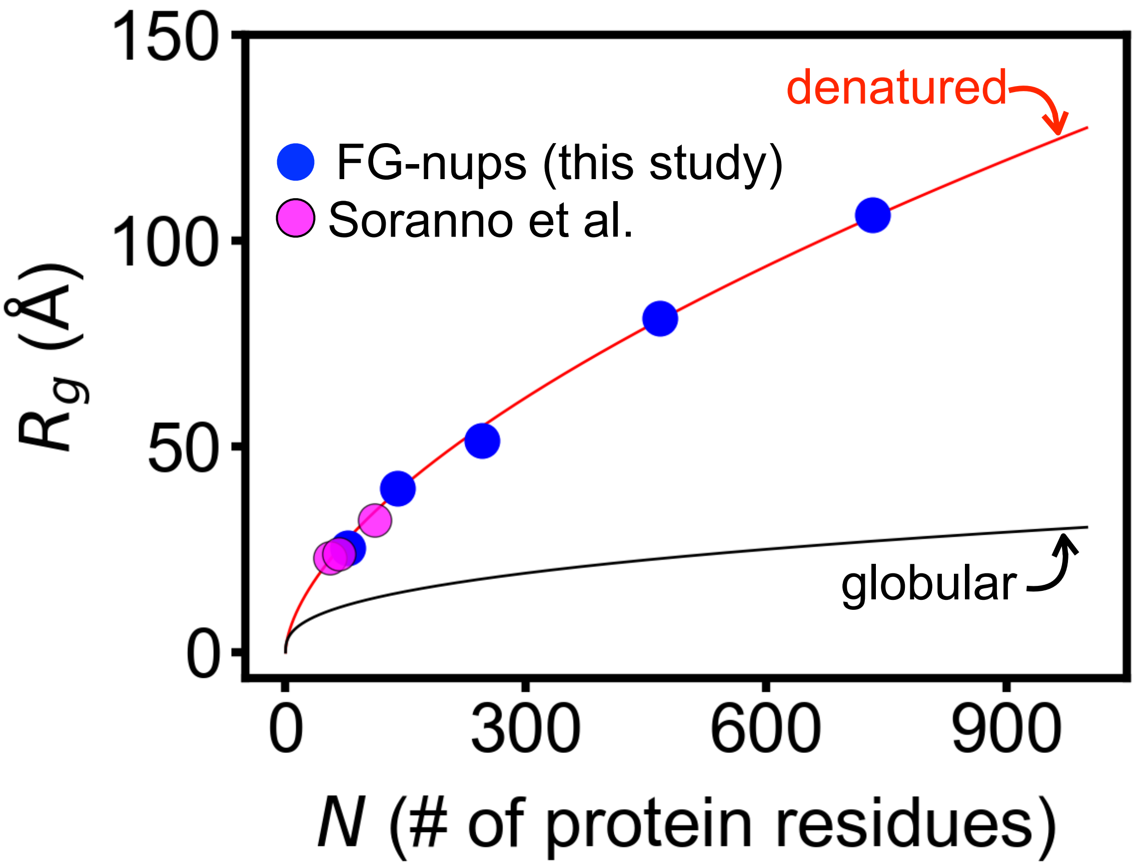

Supplementary Fig. 17: Scaling behavior of the FG-nups radius of gyration. Average radius of gyration, R_{g}, of individual FG-nup species (blue circles) is plotted as a function of the number of amino acid residues, N, comprising the FG-nups. The data were obtained by averaging the R_{g} values over the course of our CG simulations of the Lin2016 structure. Magenta circles show the R_{g} values experimentally measured by Soranno et al. [7] for chemically-denatured proteins, which have negligible internal friction. The black curve shows a scaling relation for the R_g of globular proteins [8] : $R_g(N) = 2.2N^{0.38}$. The red curve shows a scaling relation for the R_g of denatured proteins [9]: $R_g(N) = 2.02N^{0.60}$. These scaling data indicate that, within the limits of our CG model, the FG-nups can be considered to have negligible internal friction.

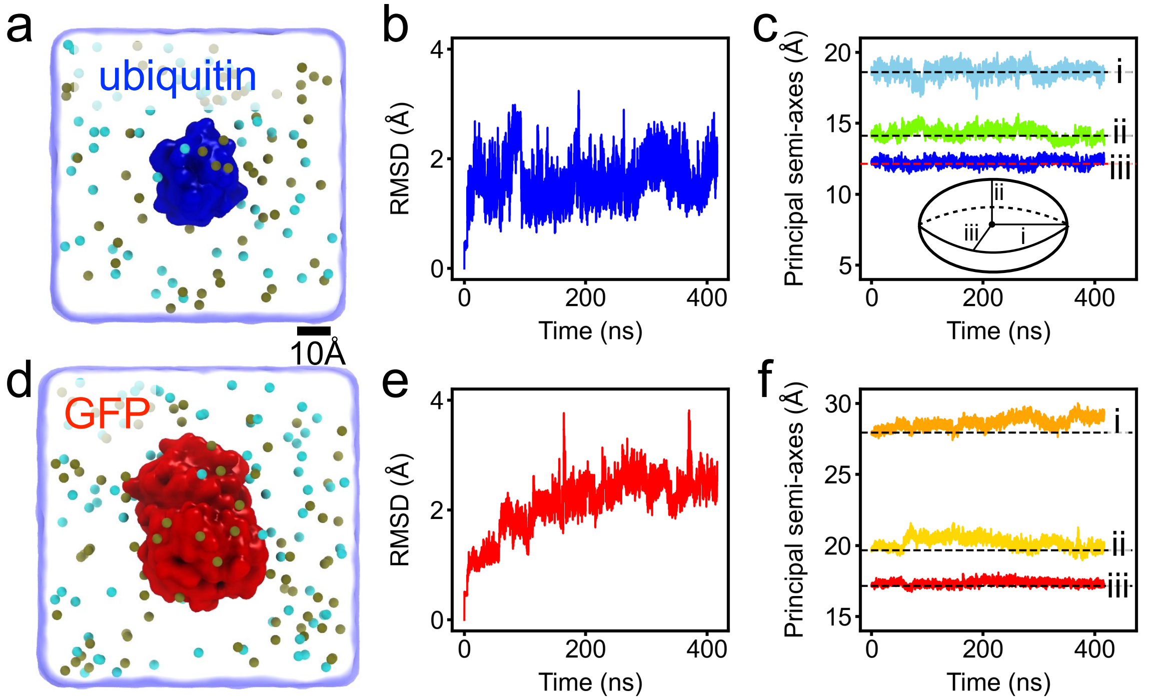

Supplementary Fig. 18: Structural fluctuations of globular proteins in all-atom MD simulations. **a** All-atom representation of ubiquitin (blue molecular surface) solvated in explicit water (semi-transparent blue) and ions (cyan and gold spheres). **b** Root mean-squared deviation of ubiquitin C_α coordinates with respect to the crystal structure values (PDB ID: 1UBQ) as a function of simulation time. **c** Principal semi-axes of ubiquitin as a function of simulation time. Inset illustrates the principal semi-axes of an ellipsoid, denoted {i,ii,iii}. Dashed lines indicate the corresponding principal semi-axes values for the rigid-body protein. **d–f** Same as in panels a–c but for a GFP protein, PDB ID 1EMA.

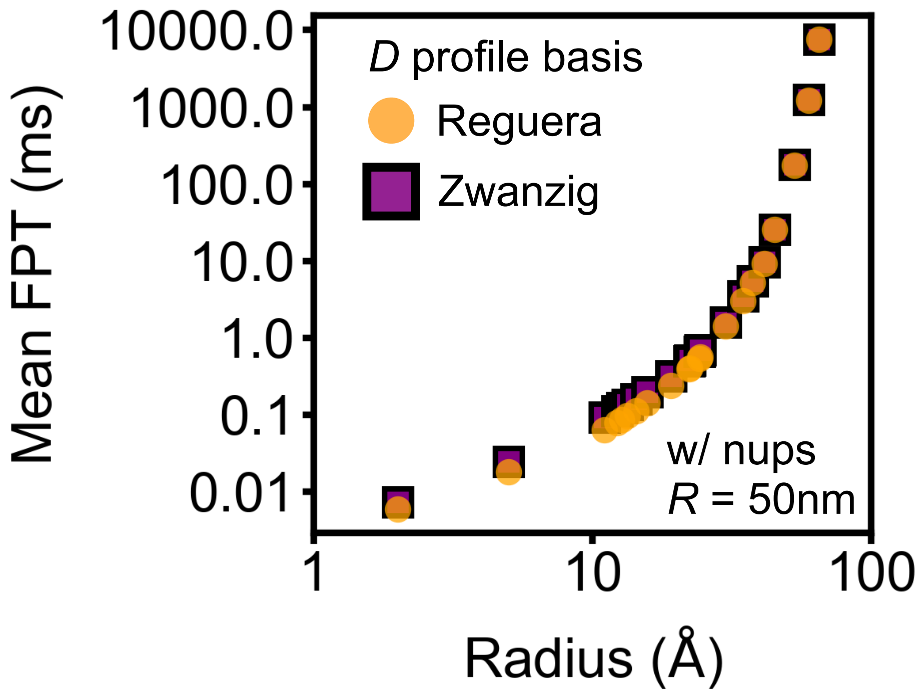

Supplementary Fig. 19: Effect of local diffusion model on Fokker-Planck MFPT. The first-passage time is plotted versus the probe radius for the Lin2016 NPC system with FG-nups present under a 50 nm-radius cylindrical confinement. The Fokker-Planck calculations were done using the local diffusion models proposed by Reguera and Rubi [10] (orange) and Zwanzig [11] (purple).

Name	PDB ID	Molecular mass (kDa)	Protein radius (Å)	Average diffusion coefficient (Å^2/ns)
Insulin	2HIU	5.812	12.61 ± 2.32	15.218
Aprotinin	4PTI	6.524	12.88 ± 4.02	14.816
Z-domain	2SPZ	6.638	13.07 ± 5.07	14.279
Ubiquitin	1UBQ	8.565	14.72 ± 2.70	13.721
Thioredoxin	1F6M	11.654	15.94 ± 2.38	12.456
α-lactalbumin	1F6S	14.066	17.08 ± 4.61	11.540
GFP	1EMA	25.379	21.13 ± 4.60	9.462
PBP	2ABH	34.421	23.38 ± 8.34	8.339
MBP	1ANF	40.570	25.18 ± 6.70	7.949
Enolase	6ENL	46.623	26.61 ± 5.70	7.640
Hemoglobin	4HHB	61.477	29.85 ± 4.16	6.933
RAR	1G5Y	102.044	35.94 ± 9.04	5.689
GAPDH	1U8F	143.471	40.94 ± 1.62	5.166

Supplementary Table 1: Rigid-body approximation of protein size and diffusion. Starting from their crystal structures, all-atom models of the proteins were constructed to include all hydrogens atoms and used to calculated the total mass. To determine the radius of a protein, we first computed the protein's moments of inertial, I_{X}, I_{Y}, and I_{Z}. We then equated the moments to those of a constant-density ellipse, $I_{\mathrm{X}} = \frac{1}{5}m(b^2 + c^2)$, $I_{\mathrm{Y}} = \frac{1}{5}m(a^2 + c^2)$ and $I_{\mathrm{Z}} = \frac{1}{5}m(a^2 + b^2)$, where m is the total mass of the protein, and determine the length of the three semi axes a, b and c. The protein radius was determined by equating the volume of a sphere to the volume of the ellipse, i.e., as $(abc)^{1/3}$. The standard deviation of the protein radius reported in the table is the standard deviation of a, b and c from that radius. The average diffusion coefficient was calculated by averaging the diagonal elements of the diffusion matrix returned for each protein by the HYDROPRO server.

Void probe radius (Å)	Diffusion coefficient (Å^2/ns)	Equivalent molecular mass (kDa)
2.0	83.304	0.071
5.0	35.740	0.709
11.043	17.192	5.183
12.253	15.618	6.729
12.75	15.056	7.436
13.352	14.428	8.349
14.314	13.530	9.943
15.708	12.416	12.557
19.051	10.390	20.386
22.162	9.036	29.806
22.395	8.948	30.600
24.1425	8.348	36.956
24.384	8.272	37.890
30.044	6.822	64.000
34.745	5.964	92.200
37.5	5.558	111.675
41.318	5.082	142.463
45.0	4.698	176.525
52.94	4.042	265.482
59.5	1.815	355.994
65.0	1.672	444.498

Supplementary Table 2: The radius and the diffusion constants of each spherical probe used for void analysis and Fokker-Planck calculations. To enable direct comparison of the CG simulation results with the void analysis data, each spherical probe was assigned an equivalent molecular mass using the best fit power-law dependence as shown in (Supplementary Fig. 9).

FG-nup species	Kim2018 copy #	Kim2018 length	Kim2018 domain	Kim2018+ copy #	Kim2018+ length	Kim2018+ domain	Huang2020 copy #	Huang2020 length	Huang2020 domain
Nsp1	48	620	1–620	48	620	1–620	48	601	1–601
Nup159	16	760	1–760	16	760	382–1141	16	695	388–1082
Nup116	16	760	1–760	16	760	1–760	16	966	1–966
Nup100	16	560	1–560	16	560	1–560	16	801	1–801
Nup49	32	220	1–220	32	220	1–220	32	270	1–270
Nup57	32	220	1–220	32	220	1–220	32	287	1–287
Nup145N	16	220	1–220	16	220	1–220	16	426	1–426
Nup1	8	720	1–720	8	720	1076–357	16	876	1076–201
Nup60	16	120	1–120	16	120	539–420	16	189	539–351
Nup42	–	–	–	8	382	1–382	8	382	1–382
Nup2	–	–	–	16	720	1–720	16	720	1–720
linker Nup100	–	–	–	16	225	561–815	–	–	–
linker Nup116	–	–	–	16	205	761–965	–	–	–
linker Nup145N	–	–	–	16	238	221–458	–	–	–

Supplementary Table 3: FG-nup domains of the Kim2018, Kim2018+ and Huang2020 NPC models. We provide the copy number, length and range of amino acids present for the domains of FG-nups included in three separate NPC models. "Huang2020" refers to the NPC model built by Huang et al. in Ref 3. Note, for Kim2018+ and Huan2020, FG-nups Nup1 and Nup60 are restrained on their N-terminal end. Linker 'connector' domains of Nup100, Nup116, Nup145N are only included in the Kim2018+ model.

Supplementary Methods: All-atom MD simulations of ubiquitin and GFP.

Two proteins were selected to be simulated with all-atom molecular dynamics (MD) in explicit solvent: ubiquitin (PDB ID: 1UBQ [12]) and GFP (PDB ID: 1EMA [13]). The *psfgen* tool was used to add any missing atoms to the 76-residue (ubiquitin) and 225-residue (GFP) proteins. The proteins were placed at the origin of separate cubic boxes measuring $78 \times 78 \times 78$ Å^3 (ubiqutin) and $86 \times 86 \times 86$ Å^3 (GFP) to ensure a buffer length of at least 30 Å between protein periodic images. The volumes were filled with pre-equilibrated water molecules using VMD. The systems were then neutralized, and ions were added to achieve a 200 mM concentration of KCl. Systems contained 48,472 atoms (ubiquitin) and 64,103 atoms (GFP), initial conformations shown in (Supplementary Fig. 18a, d).

All MD simulations were performed using the CHARMM36 [14] force field for protein, the TIP3P water model, and custom CUFIX [15] corrections for non-bonded interactions between charged groups. In all simulations, we employed 2-2-6 multiple time stepping and periodic boundary conditions, with long-range electrostatics were calculated using the Particle Mesh Ewald [16] method over an 11 Å-spaced grid. An 8-10-12 Å cutoff scheme was used to calculate van der Waals and short-range electrostatic forces. Covalent bonds to hydrogen in water and in non-water molecules were constrained using SETTLE [17] and RATTLE [18] algorithms, respectively. The Nosé-Hoover Langevin piston [19] was used to maintain constant pressure, and the Langevin thermostat to maintain a constant temperature (with a damping coefficient of 0.5 ps^{-1}). Systems were minimized using the conjugated gradient method for 1200 steps followed by a 4.8-ns equilibration simulation during which harmonic

restraints were applied to the carbon alpha atoms ($k = 1$ kcal mol^{-1} Å^{-2}). Minimization, restrained equilibration, and unrestrained production MD simulations of 400 ns were performed using NAMD2 [20], with a 2-fs integration timestep in the constant number of particles N, pressure P (1.0 atm) and temperature T (298.15 K) ensemble. Coordinates of the system were saved every 19.2 ps.

Supplementary References

1. Ghavami, A., Veenhoff, L. M., van der Giessen, E. & Onck, P. R. Probing the disordered domain of the nuclear pore complex through coarse-grained molecular dynamics simulations. *Biophysical journal* **107**, 1393–1402 (2014).

2. Peyro, M., Soheilypour, M., Ghavami, A. & Mofrad, M. R. Nucleoporin's like charge regions are major regulators of FG coverage and dynamics inside the nuclear pore complex. *PLoS One* **10**, e0143745 (2015).

3. Huang, K., Tagliazucchi, M., Park, S. H., Rabin, Y. & Szleifer, I. Nanocompartmentalization of the nuclear pore lumen. *Biophys. J.* **118**, 219–231 (2020).

4. Kim, S. J. *et al.* Integrative structure and functional anatomy of a nuclear pore complex. *Nature* **555**, 475 (2018).

5. Timney, B. L. *et al.* Simple rules for passive diffusion through the nuclear pore complex. *J. Cell Biol.* **215**, 57–76 (2016).

6. Popken, P., Ghavami, A., Onck, P. R., Poolman, B. & Veenhoff, L. M. Size-dependent leak of soluble and membrane proteins through the yeast nuclear pore complex. *Mol. Biol. Cell* **26**, 1386–1394 (2015).

7. Soranno, A. *et al.* Quantifying internal friction in unfolded and intrinsically disordered proteins with single-molecule spectroscopy. *Proc. Natl. Acad. Sci. U. S. A.* **109**, 17800–17806 (2012).

8. Skolnick, J., Kolinski, A. & Ortiz, A. R. MONSSTER: a method for folding globular proteins with a small number of distance restraints. *J. Mol. Biol.* **265**, 217–241 (1997).

9. Ding, F., Jha, R. K. & Dokholyan, N. V. Scaling behavior and structure of denatured proteins. *Structure* **13**, 1047–1054 (2005).

10. Reguera, D. & Rubí, J. M. Kinetic equations for diffusion in the presence of entropic barriers. *Phys. Rev. E* **64**, 061106 (2001).

11. Zwanzig, R. Diffusion past an entropy barrier. *J. Phys. Chem.* **96**, 3926–3930 (1992).

12. Vijay-Kumar, S., Bugg, C. E. & Cook, W. J. Structure of ubiquitin refined at 1.8å resolution. *J. Mol. Biol.* **194**, 531–544 (1987).

13. Ormö, M. *et al.* Crystal structure of the aequorea victoria green fluorescent protein. *Science* **273**, 1392–1395 (1996).

14. Huang, J. & MacKerell Jr, A. D. Charmm36 all-atom additive protein force field: Validation based on comparison to nmr data. *J. Comput. Chem.* **34**, 2135–2145 (2013).

15. Yoo, J. & Aksimentiev, A. New tricks for old dogs: improving the accuracy of biomolecular force fields by pair-specific corrections to non-bonded interactions. *Phys. Chem. Chem. Phys.* **20**, 8432–8449 (2018).

16. Essmann, U. *et al.* A smooth particle mesh ewald method. *J. Chem. Phys.* **103**, 8577–8593 (1995).

17. Miyamoto, S. & Kollman, P. A. SETTLE: An analytical version of the SHAKE and RATTLE algorithm for rigid water molecules. *J. Comput. Chem.* **13**, 952–962 (1992).

18. Andersen, H. C. Rattle - a velocity version of the shake algorithm for molecular-dynamics calculations. *J. Comput. Phys.* **52**, 24–34 (1983).

19. Feller, S. E., Zhang, Y., Pastor, R. W. & Brooks, B. R. Constant pressure molecular dynamics simulation: The langevin piston method. *J. Chem. Phys.* **103**, 4613–4621 (1995).

20. Phillips, J. C. *et al.* Scalable molecular dynamics on CPU and GPU architectures with NAMD. *J. Chem. Phys.* **153**, 044130 (2020).
