# Supplementary material for: Percolation transition prescribes protein size-specific barrier to passive transport through the nuclear pore complex
Source: Nat Commun. 2022 Sep 1;13:5138. doi: 10.1038/s41467-022-32857-1 (PMC9437005; doi:10.1038/s41467-022-32857-1)
Supplement: Supplementary file 3 — Description of Additional Supplementary Files [file 41467_2022_32857_MOESM3_ESM.pdf]

Description of Additional Supplementary Files

File Name: Supplementary Movie 1
Description: Equilibration simulation of the nuclear pore complex. The animation illustrates the last 1,000 microseconds of a 7,500- microsecond CG simulation. Colors identify the NPC scaffold (cyan), envelope (green), and tethered FG-nups (many colors). FGnups are colored by species.

File Name: Supplementary Movie 2
Description: Coarsegrained simulation of passive diffusion. The animation illustrates a 750- microsecond fragment of a CG simulation where α-lactalbumin attempts to and eventually crosses through the NPC. The crossing even happens toward the end of the animation. Colors identify the NPC scaffold (cyan), envelope (green), tethered FG-nups (white) and αlactalbumin (orange). The cylindrical confinement potential, radius of 50 nm and height of 120 nm, is outlined in black. Lower-left black scale bar, 10 nm.

File Name: Supplementary Movie 3
Description: : Illustration of a typical crossing event. The animation illustrates a 6-microsecond fragment of a much longer MD trajectory where a maltose-binding protein is seen to pass through the NPC. Colors identify the NPC scaffold (cyan), envelope (green), tethered FG-nups (white) and maltosebinding protein (red). The instantaneous coordinates of the FG-nups and of the protein were averaged over two consecutive frames (20 ns) to smooth the representation of their motion. The cylindrical confinement potential, radius of 25 nm and height of 120 nm, is outlined in black. Lower-left black scale bar, 10 nm.
