# Supplementary material for: Percolation transition prescribes protein size-specific barrier to passive transport through the nuclear pore complex
Source: Nat Commun. 2022 Sep 1;13:5138. doi: 10.1038/s41467-022-32857-1 (PMC9437005; doi:10.1038/s41467-022-32857-1)
Supplement: Supplementary file 7 — Reporting Summary [file 41467_2022_32857_MOESM7_ESM.pdf]

nature portfolio

Corresponding author(s): Aleksei Aksimentiev

Last updated by author(s): Jun 26, 2022

Reporting Summary

Nature Portfolio wishes to improve the reproducibility of the work that we publish. This form provides structure for consistency and transparency in reporting. For further information on Nature Portfolio policies, see our Editorial Policies and the Editorial Policy Checklist.

Statistics

For all statistical analyses, confirm that the following items are present in the figure legend, table legend, main text, or Methods section.

n/a	Confirmed	
☐	☒	The exact sample size (*n*) for each experimental group/condition, given as a discrete number and unit of measurement
☒	☐	A statement on whether measurements were taken from distinct samples or whether the same sample was measured repeatedly
☒	☐	The statistical test(s) used AND whether they are one- or two-sided<br>*Only common tests should be described solely by name; describe more complex techniques in the Methods section.*
☒	☐	A description of all covariates tested
☐	☒	A description of any assumptions or corrections, such as tests of normality and adjustment for multiple comparisons
☐	☒	A full description of the statistical parameters including central tendency (e.g. means) or other basic estimates (e.g. regression coefficient) AND variation (e.g. standard deviation) or associated estimates of uncertainty (e.g. confidence intervals)
☒	☐	For null hypothesis testing, the test statistic (e.g. *F*, *t*, *r*) with confidence intervals, effect sizes, degrees of freedom and *P* value noted<br>*Give P values as exact values whenever suitable.*
☒	☐	For Bayesian analysis, information on the choice of priors and Markov chain Monte Carlo settings
☒	☐	For hierarchical and complex designs, identification of the appropriate level for tests and full reporting of outcomes
☒	☐	Estimates of effect sizes (e.g. Cohen's *d*, Pearson's *r*), indicating how they were calculated

Our web collection on statistics for biologists contains articles on many of the points above.

Software and code

Policy information about availability of computer code

Data collection	ARBD (version Mar 2 1 Beta, open source), NAMD (version 2 .13, open source), HYDROPRO (linux version 10, open source)
Data analysis	Python (version 3.6.13, open source), VMD (version 1.9.3, open source). The source code and the application examples for the void analysis method and numerical solution of the 1D Fokker-Planck equation are available at https://gitlab.engr.illinois.edu/tbgl/pubdata/void_analysis and https://gitlab.engr.illinois.edu/tbgl/pubdata/fokker_planck_1d, respectively.

For manuscripts utilizing custom algorithms or software that are central to the research but not yet described in published literature, software must be made available to editors and reviewers. We strongly encourage code deposition in a community repository (e.g. GitHub). See the Nature Portfolio guidelines for submitting code & software for further information.

Data

Policy information about availability of data

All manuscripts must include a data availability statement. This statement should provide the following information, where applicable:

- Accession codes, unique identifiers, or web links for publicly available datasets
- A description of any restrictions on data availability
- For clinical datasets or third party data, please ensure that the statement adheres to our policy

The files needed to setup an annealing NPC simulation and a single-protein translocation simulation as were performed in this study have been deposited in the UIUC data bank under accession code IDB-3813848 (https://databank.illinois.edu/datasets/IDB-3813848? code=zBHYOpfySZ2zbZ6TGxXiCWzO1q1dkaWw2KF7HsMdc7A). The simulation trajectory files and scaffold structures generated in this study have been deposited in the UIUC data bank under accession code IDB-5581194 (https://databank.illinois.edu/datasets/IDB-558119}). The data presented in Figs. 1e, 2e--h, 3e, 4b--d, 4f & g, 4i & j, 5a--d, 5f, 5h--j, 6c--f are provided as a Source Data file (``NatComm_extraData.xlsx''). The analysis scripts are available upon reasonable request. And the following structures were used from the Protein Data Bank: PDB IDs 2HIU, 4PTI, 2SPZ, 1UBQ, 1F6M, 1F6S, 1EMA, 2ABH, 1ANF, 6ENL, 4HHB, 1G5Y, 1U8F.

Field-specific reporting

Please select the one below that is the best fit for your research. If you are not sure, read the appropriate sections before making your selection.

☒ Life sciences ☐ Behavioural & social sciences ☐ Ecological, evolutionary & environmental sciences

For a reference copy of the document with all sections, see nature.com/documents/nr-reporting-summary-flat.pdf

Life sciences study design

All studies must disclose on these points even when the disclosure is negative.

Sample size	Sample size was not predetermined based on statistical measures. At least two independent replicas were run for each simulation system.
Data exclusions	The mean first-passage time of the largest simulated protein (GAPDH, 144 kDa) with FG-nups present within a confinement volume of radius R=50nm was excluded from Figs. 2h, 3e, 5b, 5d. We made this decision because only one translocation event occurred (see the purple curves in Fig. 2d).
Replication	For each protein simulated (13 in total), under each condition (no nups with R=25nm; no nups with R=50nm; with FG-nups R=25nm; with FG-nups R=50nm), two independent replicas were performed, differing only the initial position of the center-of-mass of the rigid-body protein.
Randomization	Randomization is not relevant for our study as no animals or human subjects were used.
Blinding	Blinding was not relevant to this study.

Reporting for specific materials, systems and methods

We require information from authors about some types of materials, experimental systems and methods used in many studies. Here, indicate whether each material, system or method listed is relevant to your study. If you are not sure if a list item applies to your research, read the appropriate section before selecting a response.

Materials & experimental systems

n/a	Involved in the study	
☒	☐	Antibodies
☒	☐	Eukaryotic cell lines
☒	☐	Palaeontology and archaeology
☒	☐	Animals and other organisms
☒	☐	Human research participants
☒	☐	Clinical data
☒	☐	Dual use research of concern

Methods

n/a	Involved in the study	
☒	☐	ChIP-seq
☒	☐	Flow cytometry
☒	☐	MRI-based neuroimaging
